# *‘It is an entrustment’*: Broad consent for genomic research and biobanks in sub‐Saharan Africa

**DOI:** 10.1111/dewb.12178

**Published:** 2017-10-23

**Authors:** Paulina Tindana, Sassy Molyneux, Susan Bull, Michael Parker

**Keywords:** biobanks, broad consent, community engagement, Africa, trust, ethics

## Abstract

In recent years, there has been an increase in the establishment of biobanks for genetic and genomic studies around the globe. One example of this is the Human Heredity and Health in Africa Initiative (H3Africa), which has established biobanks in the sub‐region to facilitate future indigenous genomic studies. The concept of ‘broad consent’ has been proposed as a mechanism to enable potential research participants in biobanks to give permission for their samples to be used in future research studies. However, questions remain about the acceptability of this model of consent. Drawing on findings from empirical research about the role of trust in decision‐making, we argue that an account of entrustment may be an appropriate way of addressing current challenges of seeking consent for biobank research in Africa. We propose a set of key points to consider that can support the proposed entrustment framework.

## 1 INTRODUCTION

In recent years, there has been an increase in the establishment of biobanks for genetic and genomic studies around the globe. These scientific resources are broadly defined as a ‘collection of human biological specimens for research purposes’ and are seen as a public good for addressing important research questions.1,, 2 Collections in a biobank may involve blood, saliva, urine, DNA samples and other human body tissues and fluids. While most existing biobanks are in high‐income‐countries (HICs), there is a growing effort to establish biobanks in low and middle‐income countries (LMICs) to promote indigenous research. One example of this is the Human Heredity and Health in Africa Initiative (H3Africa), which has established biorepositories to facilitate future research in Africa.^3^ The three H3Africa biorepositories, in Uganda, Nigeria and South Africa, are expected to provide a platform for strengthening genomic capacity and facilitating important research projects on the continent.

There is a growing acknowledgement that genomic research and the establishment of biobanks present several ethical and practical challenges to existing ethics and regulatory frameworks. These challenges include identifying the most appropriate approach for seeking consent for reusing human biological materials, benefit‐sharing, privacy, ownership, donor involvement in decisions about future uses of samples and the role of ethics committees in such decisions.4,, 5,, 6,, 7 One of the most debated of these issues is the concept and practice of ‘broad consent’ for biobanks and future uses of samples.8,, 9,, 10 In this paper, we review the concept and practice of broad consent and the various alternative models proposed in the literature. In light of an examination from empirical studies on attitudes to biobanking in Africa, we argue that an account of entrustment may be an appropriate way of addressing current challenges of seeking consent for biobank research in Africa.

## 2 WHAT IS BROAD CONSENT?

Broad consent for biobanking is usually taken to refer to consent that allows the use of human biological samples both in immediate primary research and for future as yet unknown research purposes. Broad consent might be said to fall somewhere between specific consent (for a defined research study) and blanket consent (with no restrictions on the future use of samples).^11^ Table 1 presents an overview of the different consent models proposed in the literature for genomics and biobanking research.

**Table 1:** Key concepts for consent for use of human biological samples in research (adapted from 13)

*Specific informed consent*	Allows the use of biological samples and associated data only in immediate research: forbids any future research that is not foreseen at the time of the original consent
*Partially restricted consent*	Allows the use of biological samples and associated data in specific immediate research and associated future research
*Generic /Broad Consent*	Allows the use of biological samples and associated data in specific immediate research and future research of any kind at any time, with appropriate governance processes in place.
*Multi‐layered consent*	Requires several options to be explained to the research subjects in a detailed form to allow opt in and out options.
*Dynamic Consent*	A personalised digital communication interface permitting donors to make case by case decisions about inclusion of their samples and data in future research
*Blanket consent*	Allows the use of biological samples and associated data for future research of any kind at anytime

It has been argued that broad consent is legitimate because it is consistent with current practices, it respects the autonomy of participants and the risks involved are minimal.11,, 12,, 13,, 14,, 15 Some studies have also suggested that insisting on informed consent in its strict sense would be burdensome to participants, particularly in LMICs and could undermine important research.16,, 17 Broad consent has recently been endorsed by some international guidelines and regulations such as the recent revisions of the CIOMS guidelines^18^ and the US Code of Federal Regulations also support the concept of broad consent for future uses of samples and tissues.^19^ A recent review by Tindana and de Vries suggests that there is a growing acceptance of the use of broad consent, although some research participants say that they would like to receive some information on the range of studies that their samples will be used for.^20^

The concept of broad consent and alternative approaches to consent for biobanks have been widely discussed in high‐income‐countries (HICs).21,, 22,, 23,, 24,, 25 Although there has been recent attention to this discourse in Africa within the context of international collaborative studies, questions remain about what the key elements of broad consent should be.^26^ The literature highlights growing concerns that broad consent practices do not address issues about ownership and local control of archived samples and that more is needed to protect the interests of sample donors and their communities.^27^ Some research ethics committees in Africa have also raised concerns about the appropriateness of broad consent for future uses of samples, although this is gradually changing.28,, 29 There is need for further discussions on acceptable ways of seeking consent for biobanks in the region, and ensuring the concerns raised by sample donors, communities and ethics committees are considered in those deliberations.

We conducted an empirical study in two research settings in Africa where a significant amount of biomedical research has been undertaken over the past two decades; Navrongo in Ghana and Kilifi in Kenya. The general findings have been reported elsewhere.30,, 31 We reported that while there was general support for scientific research to be conducted, the concerns highlighted included challenges with broad consent, challenges with the ethics review process and the importance of community engagement.

Our aim in this paper is to make a case for an entrustment framework for biobanks, particularly in the African context drawing on our empirical study and other studies conducted in Kilifi and Navrongo which highlights the importance of trust relationships.32,, 33,, 34 We argue that an account of entrustment [where sample donors are seen as and consider themselves to be ‘entrusting’ samples into the hands of research institutions] is a more appropriate way of addressing current challenges with consent for biobanks. The proposed ‘entrustment framework’ is based on our empirical research finding that trust plays a central role in scientific research, as well as on the uncertainties surrounding future research uses of samples. Such a framework depends on and is strongly influenced by the level of engagement between host institutions and host communities.

### 2.1 What is entrustment?

Entrustment is broadly defined as the act of ‘assigning the responsibility for doing something to someone’. In the context of research involving human biological samples, entrustment will mean participants/communities *giving over* (entrusting) samples to research institutions to advance scientific research. But this act involves not just handing over samples but also assigning or establishing responsibilities. Entrustment requires that the one to whom responsibility is entrusted is trustworthy.^35^ According to Valerie Braithwaite, when citizens and clients say they trust an institution, ‘they are declaring a belief that on average, its agents will prove to be trustworthy and that they will live up to the trust placed in them’.^36^ Thus, the key requirement here for valid entrustment is trustworthiness. To some extent we can relate this to researcher‐participant relationships in scientific research, at least in Kilifi and Navrongo, our study sites. Our study suggested that there is an ‘unspoken’ understanding that when research participants consent to blood sampling in research, they also entrust researchers with the responsibility of using samples wisely. In return, research institutions have a moral obligation to ensure that they use these samples responsibly and reciprocate by providing tangible health benefits.^37^

Our interest is in the relationship between host communities and host institutions and the increasing role of the latter as stewards of samples. The evolving nature of research and scientific understanding of diseases and the complex web of relationships involved suggest that this enterprise is no longer an individual matter. Due to the increasing collaborative nature of scientific research, stakeholders have suggested that no individual researcher should own research samples; rather research institutions with oversight from research ethics committees should remain the stewards of these samples. This suggests that the ultimate responsibilities should lie with research institutions to manage the uses of these samples appropriately bearing in mind both the scientific and cultural value of human samples. It is therefore important to focus on building trustworthy research institutions as a way of addressing the ethical issues that arise in practice. One of the implications of the entrustment model as a justification for broad consent when bioresources are established in Africa is that it is only acceptable against a background of genuine attempts to establish trustworthy institutions which have relationships of well‐founded trust with the local communities from which the samples are gathered.

**Figure 1:**
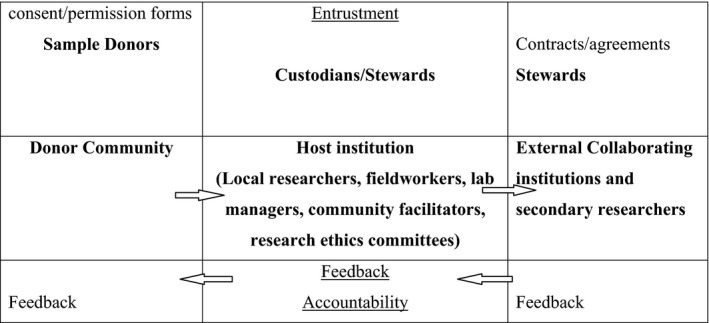
Chain of Trust in international research collaborations

Entrustment creates moral relationships between participants and researchers including the notion of responsibility. And in contrast to gift models, in an entrustment model researchers and institutions cannot do whatever they like with samples because they have responsibilities as well. Entrustment with some form of governance and accountability protects research participants and their communities from unacceptable uses of samples that may affect their wellbeing and values. It involves a chain of trust between all the key research actors (Figure 1).

Drawing on entrustment models in other discourses, such as Richardson and Belsky's partial‐entrustment model,^38^ we propose a framework that seeks to build in trustworthiness and accountability into the research enterprise. While the partial‐entrustment model and subsequent discussions specifically touch on researchers’ responsibilities to provide ancillary care39,, 40 , their key elements directly relate to how we ought to address institutions’ responsibilities in managing human biological samples. For example, the nature and strength of the engagement between researchers and participants (including participants’ vulnerability and dependency) is an important element in our proposed entrustment framework.

Another model influencing our proposal for ‘entrustment’ is a charitable trust model proposed by Winickoff and Winickoff.^41^ Here, tissues are held in trust for the donors by a trustee who oversees uses in accordance with the wishes of the benefit of the trust. This model requires the establishment of an independent party to manage the trust. The authors argue that this model would allow ‘the donor community to maximize the altruistic value of its gift’. Although this model could potentially protect the interest of sample donors, it may face challenges in its application to the research context in resource‐constrained settings such as deciding who serves on the trust and ensuring that they are adequately funded. Strengthening existing bodies such as local ethics committees and community advisory committees in these contexts could be one key way forward.

## 3 DISCUSSION

In what follows, we highlight the practical ways in which an entrustment framework can support current models of broad consent for biobanks. These include: promoting institutional trustworthiness; establishing clear institutional guidelines; strengthening consent and community engagement practices; strengthening ethics review processes; and promoting trust building as illustrated in Table 2 below.

**Table 2:** Key points to consider in an entrustment Framework

Recommendations	Points to consider
Trustworthy research institutions	Develop clear and transparent research goals Strengthen research competence Advocate for sustainable core research funds Strengthen institutional leadership Reciprocity‐ give appropriate benefits
Clear institutional guidelines	Develop institutional guidelines for sample collection Develop institutional guidelines for sample export and data sharing Develop guidelines for collaborations and access to samples and data
Effective consent and community engagement processes	Obtain informed consent for sample collection and research participation Seek entrustment for sample storage and future uses Seek entrustment for sample export and data sharing Engage community in future uses of samples
Effective and efficient research ethics committees	Develop workable standard operating procedures (SOPs) Provide adequate training Provide adequate resources Develop effective communication between REC and researchers Develop effective communication between local RECs and external RECs Actively monitor approved research
Governance	Set up a board of trustees or community advisory boards with representation from all stakeholders

Based on our analysis of empirical research and other related studies in the African context, we believe that a viable model of entrustment would need to have the following features:

## 4 PROMOTING INSTITUTIONAL TRUSTWORTHINESS

In empirical studies conducted in Kilifi and Navrongo, many researchers reported that they have been able to earn the trust of the host community by being consistent in their behaviour, being open and not going contrary to promises made to the community.42,, 43 They suggested that their closeness to local communities and their knowledge that community members have put their trust in them should motivate researchers to ‘do the right thing’. Some suggested that losing the trust of the community and access to samples – which biomedical research depends on – would be at huge cost to the research institution; even a threat to its survival. It is therefore important for host institutions to promote a culture of research integrity.

The key principles that could promote institutional trustworthiness include: good motives and intent, scientific integrity, honesty, transparency and openness, and minimizing harm and risks associated with uses of samples and data. Host research institutions should therefore establish effective mechanisms for promoting these values such as regular meetings between research teams, including field staff, and local training workshops. The research institutions and their agents (researchers, laboratory and field staff) must recognize that their trustworthiness is essential to their ability to work at all.

### 4.1 Set clear goals for research institutions

Research institutions should carry out research that addresses locally relevant health problems. This position is clearly stipulated in existing international ethics guidelines and should be taken seriously.44,, 45 The development of collaborative research proposals should be a joint effort and as much as possible should be initiated at the host institution. These goals should be shared with the various research actors within the institution and with local communities. The institution should periodically reflect on their research goals and priorities and ensure that they meet their obligations to the communities they work in. Host institutions also have a responsibility to ensure that the type of research collaborations they engage in do not leave the communities they work in disadvantaged.

### 4.2 Strengthen research competence

Following consistent calls in the literature and supported by findings from empirical research, host research institutions need sustainable personnel and infrastructure capacity to enable them to make optimum use of human biological samples locally.46,, 47 Research institutions can only achieve their research goals if they have these capacities, which would also strengthen their trustworthiness. An institutional strategic plan for strengthening local capacity is therefore important. This also suggests a set of responsibilities for funders and researchers from HICs, to try and incorporate capacity strengthening into big scientific grants. Furthermore, it requires getting clear on priority areas such as the type of personnel and equipment required for data analysis. Whenever feasible, sample export should incorporate an element of capacity building, where opportunities for local researchers to participate in sample and data analysis are provided. Capacity strengthening should include all levels of staff particularly fieldworkers (who are often community members).

### 4.3 Advocate for sustainable local core research funding

Good local research can only be possible if the host institution has adequate funding to strengthen its capacity and sustain research activities. While external funding has proved valuable in many resource‐constrained settings, the current over reliance and dependence on external funding for training of local personnel and infrastructure development is not sustainable. As authors such as Gostin have suggested, local governments should play a key role by providing adequate core funding to host research institutions.48,, 49 A recent initiative by the African Academy of Sciences to accelerate excellence in science in Africa (AESA) is a good example.^50^ Host institutions should develop effective strategies for engaging policy makers to attract sustainable local research funding and to also work towards the translation of research findings into national policies and subsequently to improve practice. Such an approach could enable research institutions to realise the social value of biomedical research.51,, 52

### 4.4 Strengthen institutional leadership

A key ingredient in sustaining scientific research in SSA is strong scientific leadership. Leadership is not only required in the technical aspects of research but also in managing human relationships within the institution and keeping members of the team motivated. In an increasingly competitive scientific environment, good leadership includes ensuring that key members of the institution at different levels have good connections with the national and international scientific community, including a strong reputation for quality work and attracting funding. These will contribute to promote trustworthiness within the research institution.

### 4.5 Reciprocity: Give‐back to the community

In addition to being trustworthy, research institutions should recognize their reciprocal obligations to host communities and develop mechanisms for returning benefits (acknowledging that individuals may not always get direct benefits from research), including the sharing of scientific knowledge to participants and wider communities. Recognising the important contribution of research participants and communities to scientific research and communicating research results back to them is a key way forward. A pragmatic way of doing this is working collaboratively with the district hospitals and health management teams to strengthen local health facilities to provide what can be much needed healthcare services.^53^ Public trust and confidence in scientific research can be bolstered if research institutions are seen to be achieving their goals and if there is also evidence of improved health in those communities such as a decline in overall morbidity and mortality. Unlike HICs settings where access to good healthcare is often available, in LICs, especially SSA, it is widely acknowledged that research institutions may have specific obligations to contribute to improving existing healthcare infrastructure in these settings.^54^

## 5 ESTABLISHING CLEAR AND TRANSPARENT INSTITUTIONAL GUIDELINES AND POLICIES

Because there is some sense of community trust in the host research institutions, it is important to have rules and oversight institutions to protect participants from effects of misplaced trust.^55^ Thus, research institutions also need clear, effective and transparent guidelines and policies on the management of human biological samples (clearly stating the conditions for sample export, storage and future uses). This should include; how samples should be collected, stored and shared between various projects in the institution, who can have access to the samples and the conditions for sample export and future uses. These should be informed by key ethical principles of respecting the rights of research participants and their communities, avoiding harm and minimizing the risks of research and maximizing benefits of research to participants and their communities.

Instituting context specific guidelines should incorporate engagement with all levels of staff, including research scientists, laboratory personnel and fieldworkers/ frontline staff, and consultation with RECs and experts in the field. Engaging with fieldworkers could be helpful in identifying some of the practical challenges in the field, especially at the collection stage and ensuring that these are adequately addressed in the guidelines. Institutional guidelines informed by community perspectives could also feed into the development of national frameworks for research involving human biological samples. Such a bottom‐up approach is important to ensure that rules and guidelines are contextually‐appropriate and implemented in practice.

## 6 STRENGTHEN CONSENT AND ENGAGEMENT WITH PARTICIPANTS AND COMMUNITY

Host institutions need to develop appropriate consent and community engagement strategies for soliciting community members’ views on acceptable future uses of samples. As Marsh et al have suggested, this could also be an important way to begin to challenge the power relations between research institutions and communities.^56^ We propose that consistent with current research ethics guidelines and regulations, specific consent should be obtained for all current studies, supported by appropriate forms of community engagement. Even with the uncertainties surrounding future uses of samples, research participants should be given the opportunity to grant permission for their samples to be exported and/or stored for future uses. But this would require some level of trust‐building as well as future engagement with the community on acceptable uses. Below are various steps to strengthen consent and engagement with participants and host research communities.

### 6.1 Obtain informed consent for sample collection and research participation

Consent is an important ethical requirement in research and thus the current practice should be to obtain informed and voluntary consent for sample collection and research participation for all defined research projects. If sample export is anticipated, this should be made clear during the consent process, including the rationales for sample export and what will happen to samples after the initial analysis process, and the need to seek broad consent for future uses of samples. Research teams should identify innovative ways of improving research participants’ basic understanding of scientific research and the value of human biological samples in research, particularly for genetic and genomic studies. As empirical studies have suggested, open research days, laboratory tours and the use of analogies and pictographs could prove very helpful in this direction.^57^ There is also the need for researchers to improve their ability to understand community priorities and concerns, and open opportunities for questions and dialogue.

Specific consent should also be obtained for sample storage and for future analyses that have been planned and anticipated. For unplanned and unanticipated future uses of samples, the current proposals for broad consent – providing the minimum information for future research purposes – are acceptable. Our position is that what is currently being described as broad consent is not informed consent in the traditional sense, but consent to something more specific ‐ use of samples within an entrustment framework which comes with a cluster of associated responsibilities and obligations. This means that research institutions have an obligation to periodically account for these samples and ensure that their future uses are supported by appropriate levels of community consultation.

The concept of entrustment is a good way of conceptualizing the transaction between research participants and researchers around future uses of samples. It goes without saying that all currently archived samples – collected without consent for future uses – should be regarded as a scientific resource *entrusted* to the host institution by the community supported by appropriate governance structures. There are obligations on researchers and research institutions to ensure that these are utilized in appropriate and culturally acceptable ways to address locally relevant health research.

Additionally, where relevant, current consent forms should include a clause requesting the permission of individual participants for samples to be stored for future research purposes and include information about the duration of sample storage. If sample and data sharing is anticipated, this information should be included in the consent forms. And as widely recommended in the literature, all future research should be approved by relevant RECs.

### 6.2 Seek entrustment for sample export and sample sharing with external collaborators

As much as possible, and taking cost implications into consideration, the host institution should build the necessary capacity to store all samples in locally established biobanks. However, sample export should not be ruled out because this practice is likely to remain a key part of research collaborations– because of scientific rationales such as the requirements for uniformity of analysis, a potential lack of local expertise. Material transfer agreements should accompany sample export and such agreements should be mutually agreed on. Secondary users of samples and data should also regard samples in their care as an entrustment, which means they have an obligation to provide appropriate updates to host institutions on the status of exported samples and ensure that they are only used for studies and analysis that have been mutually agreed upon. The MalariaGEN data release policy, which encourages local capacity building of data‐fellows in malaria‐endemic countries to enable them to use samples and data locally, is an example.^58^ Institutions should also encourage the transfer of knowledge by ensuring that local scientists get the opportunity to participate in analyses conducted abroad. There should also be appropriate recognition of local contributions in scientific publications and presentations and authorship issues should be discussed upfront to avoid any future tensions.

### 6.3 Engage with communities for future uses of samples

Effective community engagement practices are important when aiming for genuine partnerships with communities who contribute samples to determine acceptable future uses of samples. This process will be particularly important in the utilization of existing archived samples. Effective community engagement strategies should be an integral part of a research institutions’ scientific activities, and determining what counts as effective community engagement is context specific and requires some form of deliberative process.^59^ An institutional approach to community engagement may be a useful way of addressing communities’ concerns about the use of samples in research more generally. Future use of samples must be guided by a conscientious effort to engage with relevant local communities. Conducting empirical studies could also help shed light on how these types of methods would work in practice and in specific settings.

## 7 STRENGTHEN OVERSIGHT BY RESEARCH ETHICS COMMITTEES

While advocating for host institutions to bear the ultimate responsibility for the ethical conduct of their research, we acknowledge that researchers and research institutions have personal and professional interests in the conduct of research, including advancing their scientific reputation. Thus, RECs have an important role to play in providing proper oversight over these research practices. However, their roles and functions must also evolve with advancements in biomedical research. Below, we provide a set of recommendations on how RECs can become an effective component of an entrustment model for biobank research.

### 7.1 Provide adequate training to REC members

RECs require significant resources to train members to appreciate the complexities surrounding emerging scientific methodologies such as those applied in genetic and genomic research and to determine the implications of these types of research on individual participants and their communities. Regional research ethics training workshops such as those hosted by the South African Research Ethics Training Initiative (SARETI) have proved very helpful and should be sustained. Also, online courses by the US NIH and the MRC Centre for Genomics and Global Health.^60^ which aim at improving lay understanding of genetic/genomic research could prove very useful for RECs. RECs should also devise appropriate ways of obtaining expert opinion on complex scientific methodologies to facilitate the review process.

### 7.2 Provide adequate resources

Research institutions should provide adequate logistic support to enable RECs to function effectively. The need to avoid or manage conflicts of interest is important to note here. If RECs are dependent on research institutions for resources and capacity building there is the potential for them to find it difficult to make decisions about research that may not be in favour of the research institutions, particularly those that rely on research funds to sustain their operations. Nevertheless, in practice it is often only the research institutions that have the resources to do this capacity‐building. Ideally local governments should recognize the important role of RECs and fund their activities directly so that they can be independent from the research institutions in the review process. More commitment from major research funders could also go a long way to sustain capacity building activities for RECs in Sub‐Saharan Africa.

### 7.3 Develop effective communication strategies

RECs should also develop effective communication strategies to engage with researchers and understand the practical challenges encountered in the field. This could be done through periodic joint meetings and workshops within the institution or host country. Such communication will ensure that participants’ interests are respected when biorepository resources are shared. It can also facilitate the effective conduct of research for which there are plans to share data and samples, but avoiding unnecessary delays and ambiguities for researchers. RECs should also work together with the local institution to develop appropriate mechanisms for engaging with local communities as well.

In international research collaborations, multiple REC review is inevitable. Effective communication between RECs is very important to streamline the review process and avoid unnecessary delays. One approach may also be to explore joint reviews in multicentre studies to improve the efficiency of the review process.

### 7.4 Actively monitor approved research

RECs should develop effective mechanisms for actively monitoring the sharing of samples and data This could involve requesting for periodic updates from the institution on the status and use of archived samples. Observing consent processes in the field would enable RECs to understand the practical challenges being faced and take them into account in their review requirements. It is also important for RECs to be given adequate regulatory backing and power to hold institutions to account for meeting – or failing to meet ‐ their obligations to host communities.

## 8 ACTIVE TRUST‐BUILDING AND GENUINE COMMUNITY INVOLVEMENT

Responding to the ethical issues arising from sample use in research requires genuine community involvement, especially in deciding what is culturally acceptable for future use of samples stored in biobanks. Currently, the practice is to relegate this decision to the research ethics committee or a data access committee. While RECs, as independent review bodies, are appropriate for making these decisions, they could also face limitations such as the lack of expertise on genetics and genomics. Therefore, it is important for research institutions to consider setting up a board/committee of trustees to protect the interests of participants and communities. This independent advisory committee could also provide opportunities for genuine community involvement in deciding acceptable future uses of samples. Membership of this committee could include scientists, field staff, laboratory personnel and well‐informed community representatives. This committee would be responsible for advising the institution on acceptable uses of samples in research.

## 9 CONCLUSION

Despite the existence of concerns about the ethical acceptability of broad consent for biobanks and biorepositories, it is emerging as the main, and possibly only practically achievable, consent model for global health research involving the creation of bioresources. In this paper, we have argued that broad consent is an acceptable approach to consent for the collection, storage and use of biological samples as long as it is seen as consent to an entrustment i.e. the establishment of a set of agreed responsibilities and obligations. It is important for researchers and research institutions to consider the concerns of sample donors and communities in determining the appropriate use of archived human biological samples. We have argued that given the lack of consensus on the best approach to achieving high ethical standards in research using broad consent for biobanks, an approach grounded in principles of ‘entrustment’ offers a good way of establishing and maintaining trust for such research and embedding high ethical standards. We have suggested practical ways in which this entrustment framework can inform policy and practice. These include establishing trustworthy institutions, clear and transparent institutional guidelines and policies, strengthening consent and community engagement practices, strengthening ethics review and active trust building.

## CONFLICT OF INTEREST

No conflicts Declared.

## Notes

1 Kaye J. Do we need a uniform regulatory system for biobanks across Europe? European journal of human genetics: EJHG. 2006;14(2): 245‐8.

2 Gibbons SM, Kaye J**.** Governing Genetic Databases: Collection, Storage and Use. King's law journal : KLJ. 2007;18(2):1468.

3 H3Africa Consortium**.** Research capacity. Enabling the genomic revolution in Africa. Science. 2014;344(6190):1346‐8.

4 Staunton C, Moodley K**.** Challenges in biobank governance in Sub‐Saharan Africa. BMC medical ethics. 2013;14:35.

5 O'Doherty KC , Hawkins AK, Burgess MM**.** Involving citizens in the ethics of biobank research: Informing institutional policy through structured public deliberation. Social Science & Medicine. 2012;75(9):1604‐11.

6 Virani AH, Longstaff H**.** Ethical Considerations in Biobanks: How a Public Health Ethics Perspective Sheds New Light on Old Controversies. Journal of genetic counseling. 2014.

7 Upshur RE et al**.** Taking tissue seriously means taking communities seriously. BMC medical ethics. 2007;8:11.

8 Coughlin SS**.** Broad consent and biorepositories for molecular epidemiology and genomics research.International journal of molecular epidemiology and genetics. 2011;2(4):401‐2.

9 Hansson M**.** Should donors be allowed to give broad consent to future biobank research? Lancet Oncology. 2006;7:266‐9.

10 Tindana P, de Vries J**.** Broad Consent for Genomic Research and Biobanking: Perspectives from Low‐ and Middle‐Income Countries. Annu Rev Genomics Hum Genet. 2016;17:375‐93.

11 Tindana P, de Vries J**.** *op. cit. note 10*.

12 Sheehan M**.** Broad consent is informed consent. BMJ. 2011;343:d6900; author reply d1.

13 Salvaterra E et al. Banking together. A unified model of informed consent for biobanking. EMBO Rep. 2008;9(4):307‐13.

14 Petrini C**.** “Broad” consent, exceptions to consent and the question of using biological samples for research purposes different from the initial collection purpose. Social science & medicine. 2010;70(2):217‐20.

15 Helgesson G**.** In defense of broad consent. Cambridge quarterly of healthcare ethics: CQ: the international journal of healthcare ethics committees. 2012;21(1):40‐50.

16 Bull et al. Best Practices for the Ethical Sharing of Individual Health Data in Low and Middle‐income Countries. Journal of Empirical Research on Human Research Ethics 2015, Vol. 10(3) 302–313.

17 Jao I, Kombe et al. (2015). Involving research stakeholders in developing policy on sharing public health research data in Kenya: Views on fair process for informed consent, access oversight and community engagement. Journal of Empirical Research on Human Research Ethics, 10, 264‐277.

18 Revised US Code of Federal Regulations. Federal Register /Vol. 82, No. 12/Thursday, January 19, 2017/Rules and Regulations. Available from: https://www.gpo.gov/fdsys/pkg/FR-2017-01-19/pdf/2017-01058.pdf

19 Council for International Organisations of Medical Sciences. International Ethical Guidelines for Health‐related Research involving Humans. Geneva: Available from: http://cioms.ch/ethical-guidelines-2016/WEB-CIOMS-EthicalGuidelines.pdf. [Accessed on 19 May 2017]

20 Tindana P and de Vries J**.** *op. cit. note 10*.

21 Arnason V**.** Coding and consent: moral challenges of the database project in Iceland. Bioethics. 2004;18(1):27‐49.

22 Caulfield T et al**.** DNA databanks and consent: a suggested policy option involving an authorization model. BMC medical ethics. 2003;4:E1.

23 Secko DM et al**.** Informed consent in biobank research: a deliberative approach to the debate. Social Science & Medicine. 2009;68(4):781‐9.

24 Kaye J et al**.** Dynamic consent: a patient interface for twenty‐first century research networks. Eur J Hum Genet. 2015;23(2):141‐6.

25 Steinsbekk KS et al**.** We're not in it for the money‐lay people's moral intuitions on commercial use of ‘their’ biobank. Medicine, health care, and philosophy. 2011.

26 Tindana, de Vries. *op.cit* note 10.

27 Upshur et al. *op.cit note 7*.

28 de Vries J, Abayomi A, Littler K, et al. Addressing ethical issues in H3Africa research–the views of research ethics committee members. The HUGO journal. 2015;1(9):1‐4.

29 Ramsay M, de Vries J, Soodyall H, Norris SA, Sankoh O**.** Ethical issues in genomic research on the African continent: experiences and challenges to ethics review committees. Hum Genomics. 2014;8(1):15.

30 Tindana Paulina. Doctoral Thesis: Ethical issues in the collection, export, storage and uses of human biological samples in Africa: an analysis of stakeholders’ perspectives. *University of Oxford, 2013*.

31 Tindana P, Molyneux CS, Bull S, Parker M. Ethical issues in the export, storage and reuse of human biological samples in biomedical research: perspectives of key stakeholders in Ghana and Kenya. BMC medical ethics. 2014;15:76.

32 Helgesson G**.** *op.cit note 17*.

33 Bull et al. Best Practices for the Ethical Sharing of Individual Health Data in Low and Middle‐income Countries. Journal of Empirical Research on Human Research Ethics 2015, Vol. 10(3) 302–313.

34 Tindana, P et al “Seeking consent to genetic and genomic research in a rural Ghanaian setting: A qualitative study of the MalariaGEN experience.” BMC medical ethics 13(1): 15.

35 O'Neill O, NetLibrary Inc. Autonomy and trust in bioethics. Cambridge ; New York: Cambridge University Press; 2002:xi, 213 p.

36 Braithwaite V**.** Communal and exchange trust norms: their value base and relevance to institutional trust. In: Levi VBaM, editor. Trust and Governance: Russel Sage Foundation; 1998:46‐74.

37 Tindana et al. *op.cit note* 29.

38 Richardson HS, Belsky L**.** The ancillary‐care responsibilities of medical researchers. An ethical framework for thinking about the clinical care that researchers owe their subjects. *The Hastings Center report*. 2004;34(1):25‐33.

39 Richardson HS**.** Gradations of researchers’ obligation to provide ancillary care for HIV/AIDS in developing countries. *American journal of public health*. 2007;97(11):1956‐61.

40 Merritt MW**.** Health researchers’ ancillary care obligations in low‐resource settings: how can we tell what is morally required? *Kennedy Institute of Ethics journal*. 2011;21(4):311‐47.

41 Winickoff DE, Winickoff RN**.** The charitable trust as a model for genomic biobanks. *The New England journal of medicine*. 2003;349(12):1180‐4.

42 Tindana et al *op.cit note 29*.

43 Tindana et al *op.cit note 33*.

44 WMA**.** Declaration of Helsinki : ethical principles for medical research involving human subjects, October 2008. GuildfordFerney‐Voltaire: Canary ;World Medical Association; 2008.

45 Nuffield Council on Bioethics. The ethics of research related to healthcare in developing countries. London: Nuffield Council on Bioethics; 2002.

46 Tindana et al, *op.cit note 30*.

47 Ijsselmuiden CB et al**.** Evolving values in ethics and global health research. *Global Public Health. 2010;5(2):154‐63*.

48 Gostin L. The unconscionable health gap: a global plan for justice. *Lancet. 2010;375(9725):1504‐5*.

49 Gostin LO**.** A framework convention on global health: health for all, justice for all. *JAMA : the journal of the American Medical Association. 2012;307(19):2087‐92*.

50 Africa Academy of Science; Accelerating Excellence in Science in Africa (AESA): Available from: https://aasciences.ac.ke/programmes/easa/alliance-for-accelerating-excellence-in-science-in-africa-aesa/. [Accessed 7 Sept 2017].

51 Emanuel E, Wendler D, Killen J, Grady C**.** What makes clinical research in developing countries ethical? The benchmarks of ethical research. Journal of Infectious Diseases. 2004;185(5):930‐7.

52 Lairumbi GM et al**.** Promoting the social value of research in Kenya: examining the practical aspects of collaborative partnerships using an ethical framework. Social science & medicine. 2008;67(5):734‐47.

53 Molyneux S, Mulupi S, Mbaabu L, Marsh V**.** Benefits and payments for research participants: experiences and views from a research centre on the Kenyan coast. BMC medical ethics. 2012;13:13.

54 Benatar SR**.** Distributive justice and clinical trials in the Third World. Theoretical medicine and bioethics. 2001;22(3):169‐76.

55 Pellegrino ED**.** Trust and Distrust in professional Ethics. Ethics, trust, and the professions: philosophical and cultural aspects: Georgetown University Press; *1992:69‐85*.

56 Marsh V, Kamuya D, Rowa Y, Gikonyo C, Molyneux S**.** Beginning community engagement at a busy biomedical research programme: experiences from the KEMRI CGMRC‐Wellcome Trust Research Programme, Kilifi, Kenya. Social science & medicine. *2008;67(5):721‐33*.

57 Tindana et al *op.cit note 30*.

58 Parker M, Bull SJ, de Vries J, Agbenyega T, Doumbo OK, Kwiatkowski DP**.** Ethical data release in genome‐wide association studies in developing countries. PLoS medicine. 2009;6(11):e1000143.

59 Abelson J, Forest PG, Eyles J, Smith P, Martin E, Gauvin FP**.** Deliberations about deliberative methods: issues in the design and evaluation of public participation processes. Social science & medicine. 2003;57(2):239‐51.

60 Global Health Trials Network. Available from: http://globalhealthtrials.tghn.org/elearning/education/lectures/elearning-courses/introduction-to-reviewing-genomic-research. [Accessed 7 Sept 2017].

